# Evaluating Copper-Induced Oxidative Stress in Germinating Wheat Seeds Using Laser Photoacoustic Spectroscopy and EPR Techniques

**DOI:** 10.3390/toxics13070604

**Published:** 2025-07-18

**Authors:** Mioara Petrus, Cristina Popa, Ana-Maria Bratu, Alexandra Camelia Joita, Vasile Bercu

**Affiliations:** 1 National Institute for Laser, Plasma and Radiation Physics, Laser Department, 409 Atomistilor St., P.O. Box MG 36, 077125 Magurele, Romania; cristina.popa@inflpr.ro (C.P.); ana.magureanu@inflpr.ro (A.-M.B.); 2 National Institute of Materials Physics, Atomistilor 405A, 077125 Magurele, Romania; 3 Faculty of Physics, University of Bucharest, 405, Atomistilor Str., 077125 Magurele, Romania; vasile.bercu@unibuc.ro

**Keywords:** ethylene, ammonia, laser photoacoustic spectroscopy, electron paramagnetic spectroscopy, free radicals

## Abstract

Copper is an essential micronutrient for plants, but excessive levels can induce toxicity and impair physiological functions. This study evaluates the toxic effects of copper sulfate (CuSO_4_) on the germination of common wheat (*Triticum aestivum*), with emphasis on the gas emission dynamics and oxidative stress biomarkers. Seeds were germinated in agar and exposed to CuSO_4_ at concentrations of 1 µM, 100 µM, 1 mM, and 10 mM; distilled water served as the control. Ethylene and ammonia emissions were quantified using CO_2_ laser photoacoustic spectroscopy, while electron paramagnetic resonance (EPR) spectroscopy was employed to detect free radicals and Cu^2+^ complexes. Exposure to Cu concentrations ≥ 1 mM significantly inhibited germination and biomass accumulation. Enhanced ethylene and ammonia emissions, particularly at 10 mM, indicated stress-related metabolic responses. The EPR spectra confirmed the presence of semiquinone radicals and Cu^2+^ complexes under higher Cu levels. These results demonstrate that photoacoustic and EPR techniques are effective tools for the early detection of metal-induced phytotoxicity and offer a non-invasive approach to environmental toxicity screening and plant stress assessment.

## 1. Introduction

Copper (Cu) is an essential micronutrient required in trace amounts for typical physiological function in plants, but it becomes toxic when present in excess. In soils, copper’s solubility is primarily influenced by pH and organic matter content [1]. The copper requirements for healthy plant development vary among species and cultivars [2], while phytotoxicity depends on the metal’s solubility and bioavailability [3,4]. Both copper deficiency and excess can inhibit plant growth and disrupt key biochemical pathways [5,6].

Copper plays a vital role in several physiological and metabolic functions in plants. It involves photosynthesis, carbon and nitrogen metabolism, oxidative stress defense, and cell wall synthesis. Additionally, depending on its oxidation state, copper can act as a reducing or oxidizing agent in various biochemical reactions [7].

Copper deficiency, typically defined as concentrations below 5 mg/kg of a plant’s dry weight, can severely impair plant growth [2]. Symptoms initially manifest in the young leaves and reproductive organs and include an altered root architecture, leaf deformation, chlorosis, and necrosis [7,8]. Copper deficiency is also associated with a reduced chlorophyll content and impaired photosynthesis [2,7,8]; diminished activity of nitrogen-assimilating enzymes such as glutamine synthetase [9]; and altered gene expression patterns [10].

Conversely, excessive copper concentrations are toxic and elicit adverse morphological, physiological, and molecular effects throughout plant development. Numerous studies have reported reduced seed germination under copper exposure [10,11,12], although the extent of this effect varies significantly among species, indicating different tolerance levels. High copper levels also negatively impact plant morphology by reducing root and stem length and limiting leaf expansion [13,14,15,16], ultimately decreasing biomass and productivity [13,17,18]. Furthermore, excess copper can interfere with the uptake and accumulation of other essential mineral nutrients [2,14,19,20]. These toxic effects depend on the species, copper concentration, exposure duration, and growth conditions [2].

One of the most widely documented consequences of copper toxicity is the inhibition of photosynthesis, often associated with a decline in chlorophyll content [2,8,21,22,23]. This is believed to result from structural damage to the photosynthetic apparatus under elevated copper levels [2,17]. At the cellular level, copper toxicity induces oxidative stress by generating reactive oxygen species (ROS). ROS accumulation has been confirmed in multiple plant species under copper exposure [2,16,24], where it contributes to oxidative damage to proteins, lipids, and DNA, thereby disrupting cellular homeostasis.

While the adverse effects of copper stress on wheat germination and nitrogen metabolism have previously been documented [25,26], earlier studies have largely relied on traditional, often invasive techniques to assess biochemical and physiological changes. In contrast, the present study introduces an innovative, dual-analytical approach that integrates CO_2_ laser photoacoustic spectroscopy (CO_2_LPAS) and electron paramagnetic resonance (EPR) spectroscopy. CO_2_LPAS offers real-time, non-destructive detection of key volatile biomarkers (ethylene and ammonia) at ppb levels [27,28], enabling continuous monitoring of stress-induced metabolic shifts. EPR spectroscopy complements this by revealing radical species, discussing copper(II) semiquinonato [29] and Cu^2+^ accumulation patterns in distinct plant tissues with high molecular specificity. This combined strategy allows for a dynamic and comprehensive evaluation of the early-stage responses to copper toxicity in wheat, offering novel insights beyond those obtainable using the conventional methods. Compared to conventional methods such as gas chromatography (GC) and mass spectrometry (MS) [30,31,32], CO_2_LPAS enables real-time analysis without extensive sample preparation [33]. It is particularly well suited to studying dynamic biological processes, such as plant respiration. While GC and MS offer excellent chemical specificity, they are generally more time-consuming, expensive, and less adaptable to continuous or in situ monitoring. Although compact and low-cost, electrochemical sensors typically lack the sensitivity and selectivity required to detect low concentrations of gases [34,35] such as ethylene or ammonia in complex biological environments. CO_2_LPAS effectively bridges this gap, offering parts-per-billion detection limits, high selectivity via laser wavelength tuning, and the ability to monitor multiple volatile compounds simultaneously.

This study utilized CO_2_LPAS to quantify the ethylene and ammonia emissions from germinating wheat seedlings, allowing for a dynamic assessment of the physiological disruptions caused by copper toxicity. In parallel, electron paramagnetic resonance (EPR) spectroscopy was used to detect free radicals [36] and to investigate the copper uptake in wheat germ samples exposed to copper sulfate (CuSO_4_). As a highly sensitive technique for identifying species with unpaired electrons, EPR spectroscopy facilitates the analysis of paramagnetic species in biological tissues [37]. By correlating respiratory gas emissions with copper accumulation, this study seeks to offer an integrated view of the physiological and biochemical effects of copper toxicity in wheat, shedding light on the underlying stress response mechanisms. This versatile approach could also be adapted to other stressors (e.g., cadmium, drought). Against this background, our study stands out by employing an innovative dual-technique approach, leveraging CO_2_LPAS to dynamically quantify ethylene and ammonia emissions and EPR spectroscopy to characterize free radicals and copper uptake. This dual-technique approach uncovered novel mechanistic insights into copper stress: (1) the suppression of ethylene signaling likely via the inhibition of biosynthetic enzymes and (2) metabolic disruption, as evidenced by ammonia accumulation and free radical suppression. The EPR spectra also revealed tissue-specific Cu^2+^ complexation patterns, suggesting differing detoxification strategies in the roots, grains, and leaves.

## 2. Materials and Methods

### 2.1. Seed Preparation

Common wheat seeds (*Triticum aestivum*) were obtained from a local producer, a local agricultural supplier in our region. Seeds with visible defects were removed, and selected seeds were disinfected using a 3% hydrogen peroxide solution. The seeds were exposed to the hydrogen peroxide solution for a specified duration, ensuring thorough disinfection, and were then dried on absorbent paper.

### 2.2. The Germination Setup

For each treatment condition, three independent biological replicates were used. Each replicate consisted of a separate Duran glass container containing 40 individual wheat seeds germinated under identical conditions in 0.6% agar solution. This approach ensured that each replicate represented an independent biological event and that the results reflected the population-level variability rather than instrument precision. In total, 15 containers were used: 3 for each of the 5 treatment groups (control + 4 CuSO_4_ concentrations).

Gas emission measurements and EPR analyses were carried out on bulk samples from each biological replicate to obtain averaged physiological and spectroscopic responses.

### 2.3. The CuSO_4_ Treatment

Copper sulfate (CuSO_4_) solutions were prepared using copper(II) sulfate pentahydrate (Sigma-Aldrich, St. Louis, MO, USA, M = 249.99 g/mol). The concentrations tested were 1 µM, 100 µM, 1 mM, and 10 mM. Distilled water served as the control treatment. After 72 h of germination under controlled conditions (22 °C, dark), the seedlings were approximately 1 cm in size. At this stage, 2.5 mL of the respective CuSO_4_ solution or distilled water was added to each container.

### 2.4. Environmental Control

The germination process was conducted in a Nuve TK120 climate chamber (NÜVE, Ankara, Turkey, located at the National Institute for Laser Plasma and Radiation Physics, Department of Plasma Physics and Nuclear Fusion, Măgurele, Romania) at 22 °C in complete darkness for the first 72 h. After the CuSO_4_ solutions had been applied, the chamber was programmed to a 16 h light/8 h dark cycle, simulating normal photoperiod conditions. Light was provided by fluorescent lamps with a light intensity of approximately 150 µmol·m^−2^·s^−1^ at the seedling level. Relative humidity was maintained at 60 ± 5% throughout the germination and treatment periods to ensure optimal moisture levels in the agar medium.

### 2.5. Gas Analysis via CO_2_ Laser Photoacoustic Spectroscopy

**Figure 1:**
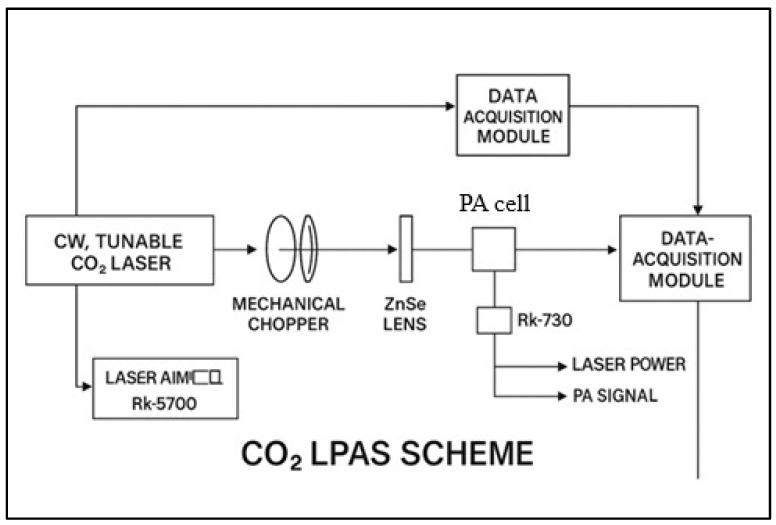
A schematic diagram of the custom-built CO_2_ laser photoacoustic spectroscopy (LPAS) system. The setup consists of three major subsystems: (1) a frequency-stabilized CO_2_ laser operating in the 9.2–10.8 μm range, (2) a longitudinally resonant photoacoustic (PA) cell designed for gas detection, and (3) a data acquisition and signal processing system, including a lock-in amplifier.

The ethylene and ammonia concentrations in plant respiration were measured 48 h post-treatment, i.e., 120 h after the start of germination. A custom-built CO_2_ laser photoacoustic spectroscopy system was used, as can be seen in Figure 1, originally developed and characterized in our earlier work [38] and later optimized for biological plant-based measurements, as described in [39], and consisting of (i) a frequency-stabilized CO_2_ laser operating in the 9.2–10.8 μm range, (ii) a longitudinal resonant photoacoustic cell with a 564 Hz resonance frequency, and (iii) an electronic signal processing unit, including a lock-in amplifier.

The sample under investigation in terms of the emission of gases produced in various biological processes is connected to the photoacoustic (PA) cell through a sealed system. The cuvettes in which the samples are placed are made of glass and equipped with an inlet and a gas outlet. For experiments that require the plant’s gas production to be recorded, the cuvettes can be adjusted to isolate these parts without destructively affecting the sample.

The pressure in the measurement setup is controlled in real time using a Type 122 A Baratron capacitive manometer (MKS Instruments, Inc., Andover, MA, USA). The emitted gas is carried by a buffer gas, in this case, synthetic air, to be introduced into the measurement cell. Before it is introduced into the FA cell, the gas sample passes through a trap that retains the interfering/ununited gas. This is practically achieved by introducing trap filters into the gas circuit, efficiently retaining any other components possibly present in the measurement gas. In this direction, a trap with a volume of 120 cm^3^ containing potassium hydroxide (KOH pellets) is necessary to remove CO_2_ and water vapor from plant respiration. After removing unwanted components, the measurement gas can be introduced into the PA cell for measurement. The gas flow was regulated using precision mass flow controllers (MKS 1179A and 2259CC, MKS Instruments, Andover, MA, USA). Nitrogen flushing between samples ensured minimal background drift (<10%).

**Figure 2:**
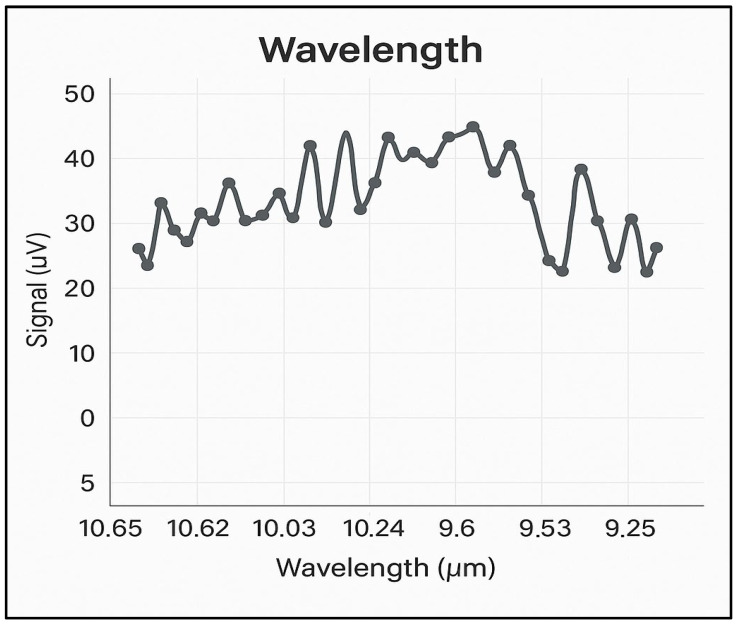
The absorption spectrum of CO_2_ laser radiation on the four branches 10P, 10R, 9P, and 9R in 6.0 pure nitrogen.

The noise encountered in the photoacoustic experiments was minimized, with the highest background noise found to be 6 µV/W or a maximum of 30 µV in nitrogen at 6.0 purity (99.9999%). Figure 2 shows the background noise spectrum in nitrogen, at atmospheric pressure, across all four branches of the CO_2_ laser emission spectrum, i.e., between 9.2 and 10.8 µm. With this noise value and the optimized characteristics of the photoacoustic cell, a detection limit below 1 ppb for ethylene at the wavelength of the 10P(14) line of the CO_2_ laser is ensured.

**Figure 3:**
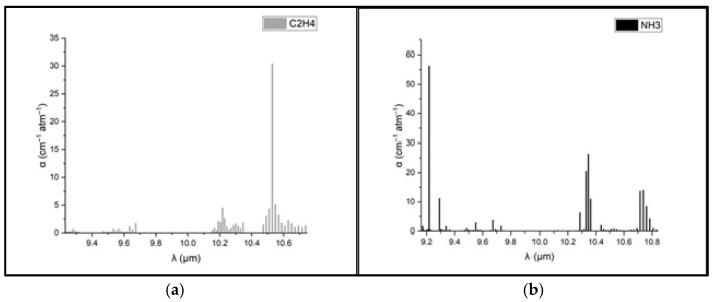
Absorption coefficients at laser lines for (**a**) ethylene and (**b**) ammonia [39,40,41].

**Figure 4:**
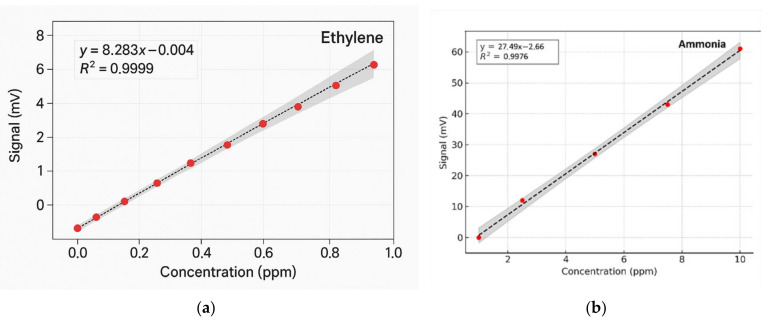
Regression curves and Pearson’s correlation coefficients using GraphPad Prism version 10 for (**a**) ethylene and (**b**) ammonia [41].

To determine the concentrations of ethylene and ammonia in the respiration of common wheat plants, the absorption coefficients for the two gases were preliminarily determined in the emission range of the CO_2_ laser. Thus, for the determination of the absorption coefficients of ethylene (see Figure 3a), a certified, commercially prepared mixture containing 0.96 ppm ethylene in pure nitrogen was used. To determine the ammonia absorption coefficients (see Figure 3b), a certified mixture containing 10 ppm (parts per million) of ammonia in pure nitrogen was used. These measurements were taken at a total pressure of approximately 1030 mbar and a temperature T = 23 °C. To ensure selective detection of ethylene and ammonia in the plant-emitted gas mixture, the CO_2_ laser was tuned to specific laser lines with known, high absorption coefficients, the 10P(14) line at λ = 10.529 µm for ethylene (α = 30.4 cm^−1^ atm^−1^) and the 9R(30) line at λ = 9.217 µm for ammonia (α = 57.12 cm^−1^ atm^−1^), based on certified standard mixtures. To eliminate potential spectral interference from common background gases such as CO_2_ and water vapor, the sample stream was passed through a trap containing potassium hydroxide (KOH pellets), which effectively removed these components before analysis. The photoacoustic cell was flushed with high-purity nitrogen (N_2_ 6.0, 99.9999%) between samples to minimize background noise and carryover. Background spectra (Figure 2) confirmed low noise and the absence of interfering absorption across the CO_2_ laser emission range. Additionally, the calibration curves for both target gases (Figure 4) confirmed the linearity and specificity of the system’s response at the chosen wavelengths.

The method’s high sensitivity allowed us to investigate some fundamental processes in plant physiology. As long as the absorption factors of ethylene and ammonia at separate laser wavelengths were specifically estimated, the CO_2_ laser was set to specific lines: first on the 10P(14) line and then on the 9R(30) line. Calibration measurements (concentration-dependent response) for both ammonia and ethylene (see Figure 4a,b) were experimentally determined using the accepted values for ethylene and ammonia.

### 2.6. The Statistical Analysis

The data are presented as the mean ± standard deviation (SD). A one-way analysis of variance (ANOVA) was used to evaluate the significance of the differences between treatment groups for each measured parameter (gas emission levels). When the ANOVA indicated significant differences (*p* < 0.05), Tukey’s post hoc test was applied to identifying specific group differences. The statistical analysis was conducted with GraphPad Prism 10 and OriginPro 2019. All statistical analyses were based on three independent biological replicates per group, ensuring a meaningful comparison of the treatment effects.

### 2.7. Paramagnetic Species Analysis via EPR Spectroscopy

Electron paramagnetic resonance (EPR) spectroscopy was employed to monitor the copper uptake from aqueous solutions with varying concentrations. This technique also enabled the detection of the free radicals present in the wheat samples.

EPR spectroscopy is a powerful analytical tool that provides unique information about magnetic interactions in atomic or molecular systems containing unpaired electrons. Its versatility allows for a wide range of applications—from studying the relaxation processes in multilevel spin systems in a crystal [42] to identifying the free radicals induced by ionizing radiation [43] and investigating cultural heritage materials [44]. It can also be used to analyze diamagnetic systems via spin-probe and spin-trapping techniques [45].

The EPR experiments were carried out at the Research Center for Advanced ESR Techniques of the National Institute of Materials Physics (Măgurele, Romania). All measurements were performed at room temperature using a Bruker EMX-plus X-band spectrometer (Bruker, Billerica, MA, USA) equipped with a Varian E-12 electromagnet [46].

EPR spectroscopy was employed to monitor the copper uptake from aqueous solutions with varying concentrations. This technique also enabled the detection of the free radicals present in the wheat samples. Due to the polar nature of the growth medium, measurements were performed on dried samples.

To investigate the absorption of copper, EPR measurements were carried out on three types of samples: agar substrate with plant roots, germinated wheat grains, and leaves. For each sample type, experimental determinations were performed at three different CuSO_4_ concentrations: 100 µM, 1 mM, and 100 mM. At concentrations lower than 1 mM, the presence of Cu ions was not observed in the EPR spectra [47,48].

**Figure 5:**
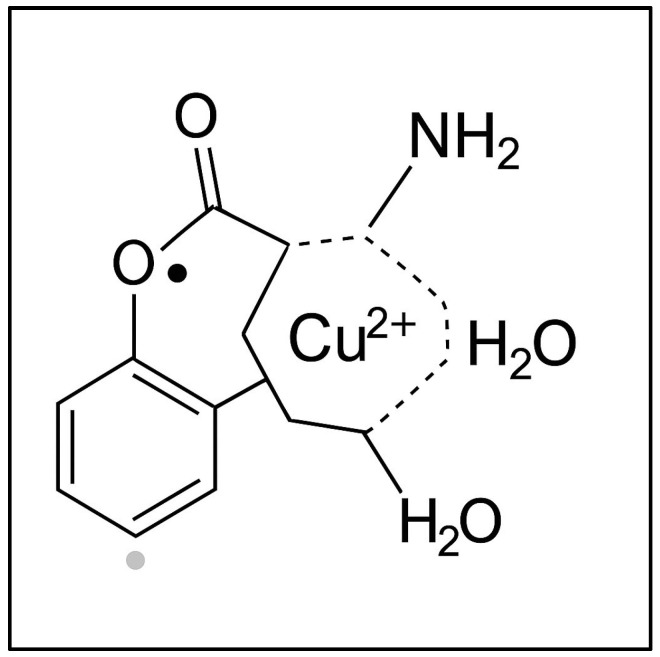
The proposed structure of a copper(II)–semiquinone radical complex with plant-derived ligands.

A common feature observed in all sample types (agar with roots, grains, and leaves) was a signal corresponding to a g-factor of g = 2.0048 ± 0.0002, also present in ungerminated wheat grains. This signal was attributed to the presence of semiquinone-type free radicals [49]. Figure 5 illustrates a coordination complex formed between a copper(II) ion (Cu^2+^) and a semiquinone-type radical ligand derived from a catecholic structure. The Cu^2+^ center is coordinated into a distorted square planar or square pyramidal geometry involving (i) two oxygen atoms from the semiquinone ligand (highlighting its bidentate binding and radical character); (ii) one amino group (representing nitrogen donor atoms from amino acids or peptides in the plant tissue); and (iii) one or two water molecules (completing the coordination sphere). This model, presented in Figure 5, reflects the interaction between transition metal ions and organic radicals observed in the wheat germ samples via EPR spectroscopy. The semiquinone radical is EPR-active, and its presence, alongside Cu^2+^, indicates the interactions during Cu exposure.

In biochemical processes, copper ions can exist in both Cu^2+^ and Cu^+^ oxidation states. The valence state plays a critical role in mediating intracellular transport and reactivity. However, due to its closed-shell electronic configuration (d^10^), Cu^+^ is EPR-silent and cannot be detected by this technique. In contrast, Cu^2+^ ions, with a d^9^ electronic configuration and a single unpaired electron (S = 1/2), produce a characteristic EPR signal. This unpaired electron interacts with the copper nucleus (I = 3/2), resulting in a typical four-line hyperfine splitting pattern.

Considering the ground-state orbital symmetry (d_x_^2^–_y_^2^) of the Cu^2+^ ion—which depends on the nature of the coordinating ligands—the EPR spectrum is expected to display a characteristic anisotropic signal with hyperfine splitting observed in the parallel region.

In cases where Cu^2+^ ions form clusters or aggregates, the EPR spectra are affected by additional exchange or dipolar interactions. Depending on their strength, these interactions can obscure the anisotropic features or even the hyperfine structure entirely.

## 3. Results

The exposure of germinating wheat seeds to increasing concentrations of copper sulfate resulted in significant physiological and biochemical alterations, as evidenced by changes in the plant respiration markers.

### 3.1. The Germination Protocol for Common Wheat Plants (Triticum aestivum)

A controlled germination protocol was carried out for common wheat seeds in a 6% agar solution, thus establishing a protocol for seed germination in a controlled environment. For three consecutive days and 48 h after the seedlings were contaminated, the samples were analyzed by measuring the concentrations of ethylene and ammonia in the respiration of the wheat plantlets, thus also establishing a protocol for the analysis of plant respiration using CO_2_LPAS. The parameters of the CO_2_LPAS system are presented in Table 1.

The respiration in common wheat plants (*Triticum aestivum*) under controlled conditions and heavy metal stress was analyzed [49] according to the protocol outlined in Table 2. The heavy metal stress was induced using CuSO_4_ solutions at concentrations of 1 µM, 100 µM, 1 mM, and 10 mM.

### 3.2. Gas Emission Analysis

**Figure 6:**
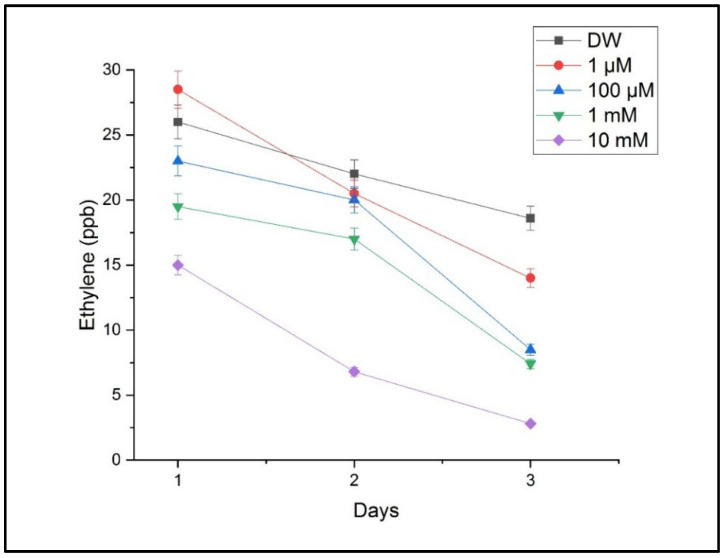
The ethylene concentrations in the headspace of germinating wheat seeds after exposure to different concentrations of CuSO_4_ compared with those for the reference sample, with DW (distilled water). Mean ethylene emission (ppmV) measured via LPAS at 120 h post-germination; error bars represent the standard deviation from three replicates.

The measurements performed using CO_2_ laser photoacoustic spectroscopy revealed significant alterations in the ethylene and ammonia concentrations in the headspace of the treated samples. Ethylene levels, which are associated with stress signaling and growth regulation, showed a progressive decrease with an increasing Cu concentration. The highest ethylene emission was detected in the seedlings treated with 10 mM of CuSO_4_, indicating a strong stress response, as shown in Figure 6.

**Figure 7:**
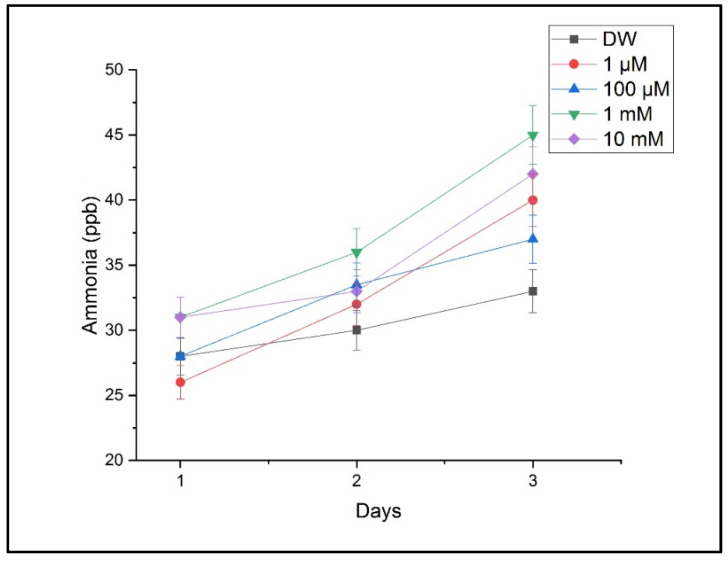
Ammonia concentration in plant respiration under varying CuSO_4_ treatments. Ammonia levels were detected using LPAS. Values correspond to mean ± SD from triplicate measurements.

Figure 7 presents ammonia levels indicative of a disrupted nitrogen metabolism. Ammonia increased significantly under the 1 mM and 10 mM treatments, supporting the hypothesis that copper interferes with key metabolic pathways.

**Figure 8:**
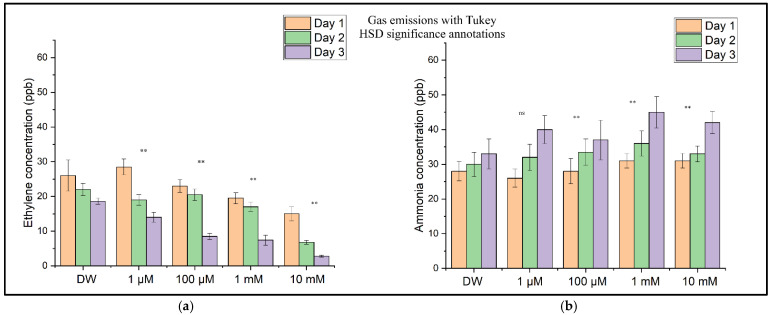
(**a**) The ethylene (C_2_H_4_) concentrations in the headspace of wheat seedlings measured on Day 1, Day 2, and Day 3 after CuSO_4_ treatment. (**b**) The ammonia (NH_3_) concentrations in the headspace of wheat seedlings were measured on the same days. Bars represent the means of three biological replicates ± SD. Significance relative to the control (DW) was assessed using Tukey’s HSD post hoc test: *p* < 0.01 (**), ns = not significant.

Figure 8 shows the concentration levels of ethylene (Figure 8a) and ammonia (Figure 8b) for the sample batches (with DW (distilled water) and CuSO_4_ solution) determined through independent measurements performed over three consecutive days. These distributions show a decrease in the ethylene concentration as the CuSO_4_ concentration increases, corresponding to inhibition of the wheat seedlings’ growth and development, followed over time at all four concentrations. In contrast to ethylene, the ammonia concentration increases with an increase in the CuSO_4_ concentration and the duration of copper sulfate solution assimilation in the seedlings. This increase in ammonia concentration corresponds to the degradation of proteins and amino acids at the plant level [26].

**Figure 9:**
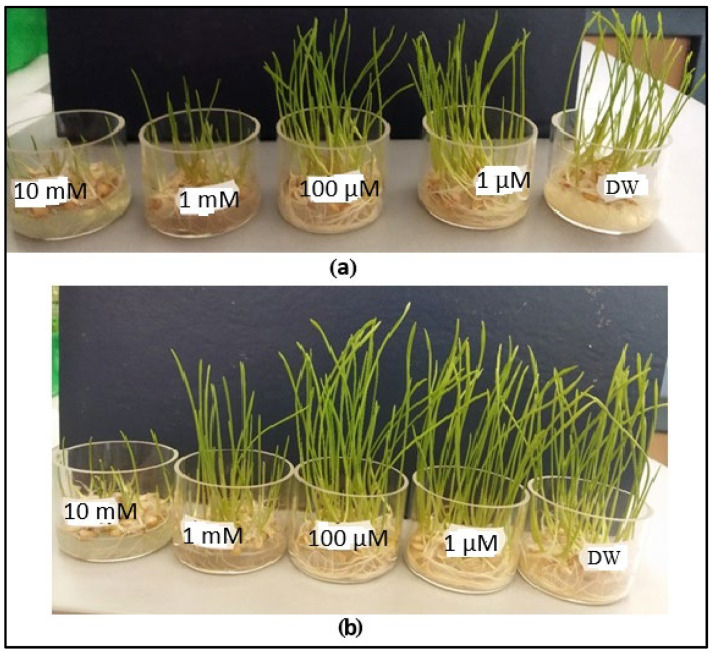
Visual representation of common wheat germ growth: (**a**) 48 h and (**b**) 72 h after exposure to distilled water (DW) and varying concentrations of CuSO_4_.

Figure 9a,b show the evolution of common wheat germs over 72 h after exposure to copper sulfate. While visual analysis reveals significant growth differences only in the germs treated with the highest concentrations (1 mM and 10 mM of CuSO_4_), germs exposed to lower concentrations (1 µM and 100 µM) appear visually similar to the distilled water control. However, our measurements using laser photoacoustic spectroscopy suggest that changes indeed occur at the cellular level, even without any visible signs of stress.

### 3.3. Statistical Analyses

A statistical analysis was conducted to evaluate the impact of CuSO_4_ exposure on ethylene (C_2_H_4_) and ammonia (NH_3_) emissions during wheat germination. The one-way ANOVA revealed significant differences among the treatment groups in their ethylene (*p* = 0.0054) and ammonia (*p* = 0.0010) concentrations. Post hoc comparisons using Tukey’s Honest Significant Difference (HSD) test demonstrated that the ethylene emissions were significantly reduced at all tested CuSO_4_ concentrations (1 µM to 10 mM) compared to those in the control group treated with distilled water (*p* < 0.01 for all comparisons). For ammonia, significantly higher emission levels were observed at concentrations of 100 µM, 1 mM, and 10 mM of CuSO_4_ (*p* < 0.01), while the 1 µM treatment did not differ significantly from the control (*p* = 0.058). These results indicate dose-dependent suppression of ethylene biosynthesis and concomitant stimulation of ammonia release under copper-induced stress, suggesting a physiological shift in metabolic responses to heavy metal exposure.

The results of the one-way ANOVA tests for the ethylene (C_2_H_4_) and ammonia (NH_3_) data are presented in Table 3. These *p*-values are <0.01, indicating statistically significant differences among the treatment groups for both gases.

**Figure 10:**
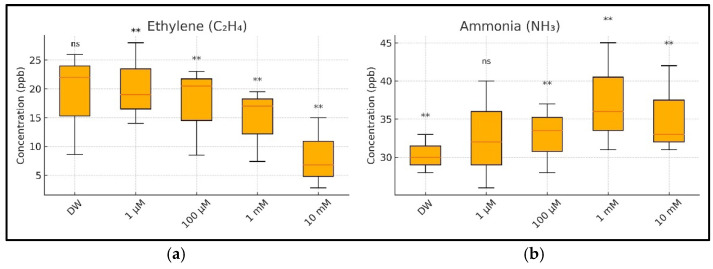
A summary of the ethylene (C_2_H_4_) and ammonia (NH_3_) concentrations in the respiration of germinating wheat seeds under copper (CuSO_4_) stress, aggregated across three measurement days (Days 1–3): (**a**) boxplot of ethylene concentrations showing a significant, dose-dependent decline with increasing CuSO_4_ concentrations; (**b**) boxplot of ammonia concentrations showing a significant increase from 100 µM upward, indicating stress-induced nitrogen metabolism disruption. Tukey’s HSD post hoc comparisons were performed relative to the control group (DW): ns = not significant, ** = *p* < 0.01. Values are based on three biological replicates per treatment.

To assess the differences between treatment groups further, Tukey’s HSD post hoc test was applied following the one-way ANOVA for both ethylene and ammonia emissions. The results of these comparisons are visually summarized in Figure 10. For ethylene (Figure 10a), all CuSO_4_ treatments showed statistically significant reductions in emissions compared to those in the control (*p* < 0.01), consistent with the dose-dependent suppression observed in the earlier figures. In contrast, ammonia levels (Figure 10b) were significantly elevated only at concentrations of 100 µM and above (*p* < 0.01), while the 1 µM treatment did not differ significantly from the control. These post hoc findings confirm that copper exposure induces opposing shifts in respiratory gas production, with ethylene biosynthesis suppressed and ammonia accumulation enhanced in a concentration-dependent manner.

### 3.4. A Comparative Analysis of the Ethylene and Ammonia Emissions on Days 1, 2, and 3

Ethylene (C_2_H_4_) and ammonia (NH_3_) emissions, measured via CO_2_ laser photoacoustic spectroscopy, showed opposing trends in response to increasing CuSO_4_ concentrations, particularly pronounced at concentrations above 100 µM. This methodological approach is consistent with that used by Popa et al. [48], who applied CO_2_ laser photoacoustic detection to monitoring the gas emissions from *Triticum aestivum* under cadmium stress.

Table 4 consolidates the data on the time evolution of the ethylene and ammonia emissions, illustrating the dynamic physiological responses of *Triticum aestivum* to varying concentrations of Cu^2+^ stress across three days. On Day 1, the onset of Cu^2+^ exposure already induced noticeable suppression of the ethylene emissions, even at the lowest concentration tested (1 µM of CuSO_4_), where a 49.1% reduction was observed. This suppression intensified with an increasing CuSO_4_ dose, reaching approximately 73% at 10 mM. Concurrently, the ammonia emissions began to exhibit an upward trend starting from 100 µM of CuSO_4,_ signaling the initiation of stress-induced metabolic adjustments, particularly at higher concentrations. By Day 2, the inhibitory effect on ethylene production became more pronounced, indicating a sustained and deepening impact of CuSO_4_ toxicity on ethylene biosynthesis or signaling pathways. Correspondingly, ammonia levels showed a sharp increase, with the concentrations at 10 mM of CuSO_4_ more than doubling compared to those for the control. This cumulative rise in ammonia provides compelling evidence of progressive metabolic disruption and heightened stress responses as the exposure time to CuSO_4_ extends. The trends observed on previous days were amplified further on Day 3. Ethylene levels demonstrated a sharp, dose-dependent decline, with the emission rates dropping by 51.0% at both 1 µM and 1 mM CuSO_4_ relative to those for the control (28.6 ppb). At the highest concentration (10 mM), ethylene emissions were suppressed by over 90%, strongly suggesting the severe disruption of essential regulatory or metabolic pathways under extreme Cu^2+^ stress, potentially linked to oxidative damage. In stark contrast, ammonia levels continued their increase proportional to the CuSO_4_ dose, escalating from 23 ppb in the control to 62 ppb at 10 mM—a substantial 169.6% rise. This significant elevation in ammonia is indicative of enhanced deamination of amino acids or widespread protein catabolism, serving as critical markers of severe metal-induced stress. The combined pattern of decreasing ethylene and increasing ammonia throughout the three days provides a robust indicator of the significant metabolic activity occurring in response to escalating copper toxicity.

### 3.5. The Detection of Free Radicals and Cu^2+^ Complexes via EPR Spectroscopy

To assess the uptake and assimilation of Cu^2+^ ions by the wheat plants, EPR measurements were performed on three distinct plant components: the roots along with agar growth medium, germinated wheat grains, and leaves. This approach allowed for comprehensive evaluation of copper’s distribution and accumulation within different parts of the plant.

**Figure 11:**
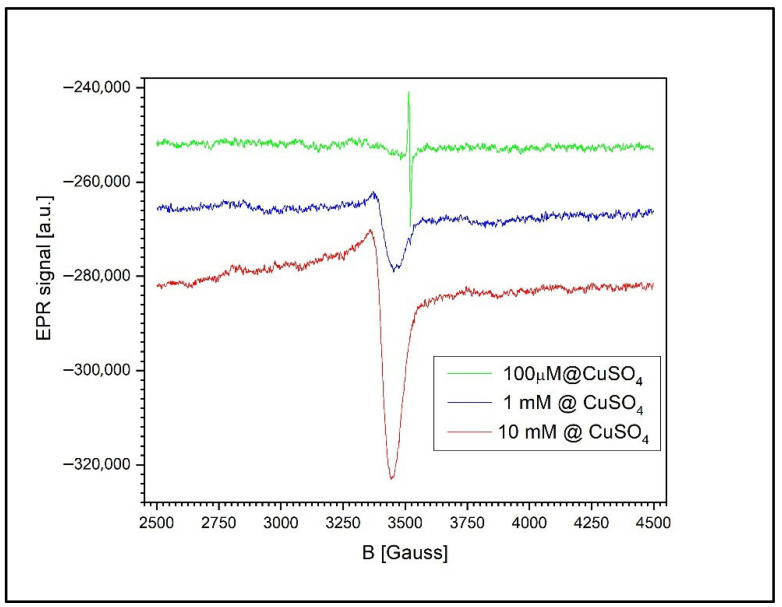
The EPR spectra of wheat roots and agar medium after treatment with CuSO_4_ at 100 µM, 1 mM, and 10 mM. A semiquinone radical signal (g = 2.0048) is present at all concentrations, while a broad Cu^2+^ signal with unresolved anisotropy appears at ≥1 mM, indicating copper accumulation and possible ion interactions.

Figure 11 presents the EPR signals recorded from the wheat roots and the surrounding agar medium at three different CuSO_4_ concentrations: 100 µM, 1 mM, and 10 mM. At concentrations below 1 mM, the spectrum reveals only the signal associated with free radicals in the roots of the germinated wheat grains, corresponding to a g-factor of g = 2.0048 ± 0.0002 and a peak-to-peak linewidth of 6.5 G.

At higher concentrations (1 mM and 10 mM), a second, asymmetric EPR signal appears, indicating the presence of unresolved anisotropy. This broadening is likely due to exchange and/or dipole–dipole interactions between the Cu^2+^ ions. The new signal is characterized by a g-factor of g = 2.0677 ± 0.0002 and a peak-to-peak linewidth of 85.5 ± 0.5 G.

At 1 mM of Cu^2+^, the free radical signal is still detectable, though significantly reduced in amplitude relative to the Cu^2+^ signal, with a Cu^2+^-to-radical amplitude ratio (I_Cu/I_RL) of 12.2. However, at 10 mM of Cu^2+^, the free radical signal is no longer observed, likely due to sensitivity limitations. In this case, the semiquinone radical signal is more than 120 times weaker than the Cu^2+^ ion’s signal and thus falls below the detection threshold.

**Figure 12:**
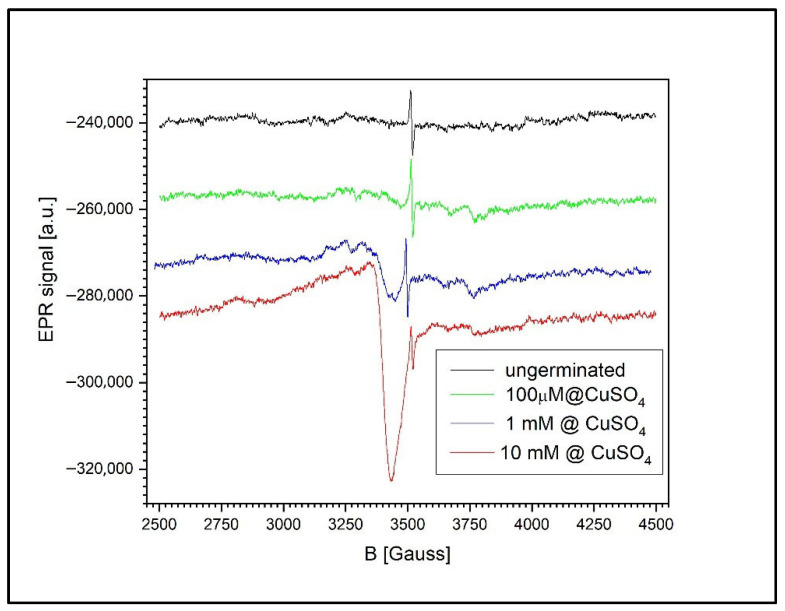
EPR spectra of ungerminated and germinated wheat grains exposed to CuSO_4_ at 100 µM, 1 mM, and 10 mM. Semiquinone radical signals are present in all samples, while Cu^2+^ signals become detectable at ≥100 µM and increase with concentration, suggesting Cu cluster formation.

Figure 12 shows the EPR signal evolution for both ungerminated wheat grains and grains germinated in CuSO_4_ solutions of varying concentrations: 100 µM, 1 mM, and 10 mM. A common feature across all samples is the presence of the semiquinone-type free radical signal, observed even in the non-germinated control grains.

For the germinated wheat grains, the EPR spectrum contains not only the characteristic semiquinone radical signal (g = 2.0048 ± 0.0002) but also additional fine structures. These secondary signals originate from various free radicals involved in the complex biochemical processes associated with germination, as previously reported in the literature [50].

The accumulation of Cu^2+^ in the wheat grains becomes detectable only at concentrations above 100 µM. The shape of the Cu^2+^ EPR signal at higher concentrations suggests the presence of large Cu^2+^ clusters, as evidenced by the absence of a resolved hyperfine structure typically observed for isolated Cu^2+^ ions. This broadening effect is likely caused by dipole–dipole or exchange interactions between closely spaced Cu^2+^ centers.

Moreover, the size and abundance of these clusters appear to increase approximately linearly with the CuSO_4_ concentration. This is reflected in the ratio of the Cu^2+^ EPR signal’s intensity to that of the semiquinone radical. At 1 mM, this ratio is 0.549, whereas at 10 mM, it rises to 5.042, indicating a substantial increase in Cu^2+^ accumulation within the grains.

**Figure 13:**
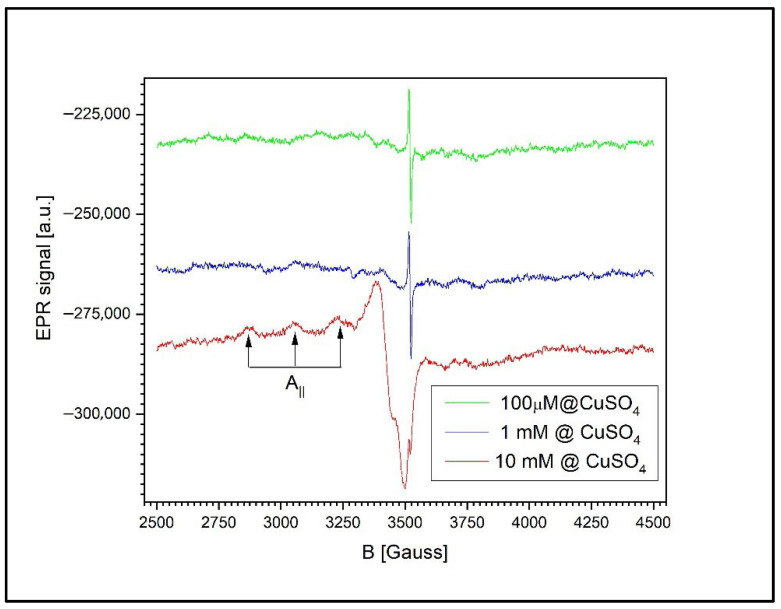
The EPR spectra of the wheat leaves exposed to CuSO_4_ at 100 µM, 1 mM, and 10 mM. The Cu^2+^ signal exhibits well-resolved hyperfine splitting, indicative of isolated ions in a tetragonally distorted environment. The Cu^2+^/semiquinone signal ratio increases with concentration, suggesting intracellular distribution without clustering.

Figure 13 illustrates the dependence of the EPR signal on the CuSO_4_ concentration in the wheat leaves. Although the spectra exhibit the same general features across all concentrations, closer inspection reveals notable differences compared to the previously analyzed samples (roots and grains). Specifically, in the case of the leaves, the EPR spectrum of Cu^2+^ displays a distinct hyperfine structure characteristic of isolated Cu^2+^ ions. This structure arises from the interaction between the magnetic moment of the unpaired electron and the nuclear magnetic moment of copper, associated with a nuclear spin of I = 3/2.

The presence of a well-resolved hyperfine structure strongly suggests that the Cu^2+^ ions in the leaf tissue are coordinated into a molecular environment of tetragonal symmetry with orthorhombic distortion. The spectral parameters characterizing this configuration are as follows:

g_ₓ_ = 2.0614 ± 0.0002,

g_ᵧ_ = 2.0267 ± 0.0002,

g_*z*_ = 2.2399 ± 0.0002,

and the hyperfine coupling constant A_*z*_ = 175 ± 2 G. Only three of the four expected hyperfine components are resolved in the spectrum, as the fourth overlaps with the signal along the g_x_; direction.

Interestingly, the relative intensity ratio between the Cu^2+^ signal and the semiquinone free radical signal remains consistent as the CuSO_4_ concentration increases. At 1 mM, the Cu^2+^/semiquinone signal ratio is 0.1367, while at 10 mM, it increases significantly to 13.59. This consistent ratio, coupled with the clearly resolved hyperfine structure, suggests that in the leaf tissue, copper ions are more uniformly distributed within the cells, rather than aggregating into clusters. These findings imply the presence of distinct mechanisms of copper’s assimilation into the leaves compared to those in the roots or grains, leading to a more homogeneous intracellular distribution of Cu^2+^ at the tested concentrations.

## 4. Discussion

This study provides new mechanistic insights into the physiological and biochemical responses of wheat seeds (*Triticum aestivum*) during early germination under copper (Cu^2+^) stress, using an integrated, non-invasive analytical approach.

The key innovation of this study lies not in identifying a new biological effect of copper stress but in employing a synergistic, non-invasive analytical platform to quantify and correlate both gaseous and paramagnetic responses in germinating wheat. By leveraging the sensitivity of CO_2_LPAS for volatile emissions and the molecular specificity of EPR for radical species and metal complexes, we were able to detect early disruptions in ethylene and ammonia dynamics and link them directly to oxidative processes and copper uptake. This integrated approach enables the detection of subtle physiological stress markers in real time and in situ, representing a significant methodological advancement over the traditional gas chromatography, spectrophotometry, and destructive tissue assays commonly used in prior studies.

A central mechanistic finding is the suppression of ethylene biosynthesis under copper exposure, as evidenced by the dose-dependent decrease in ethylene (C_2_H_4_) emissions across all tested CuSO_4_ concentrations. By Day 3, ethylene levels were reduced by over 90% at 10 mM of CuSO_4_, indicating a strong inhibitory effect. Ethylene is essential for seed germination and stress adaptation, and our data suggest that excess copper interferes with ethylene synthesis pathways—likely by inhibiting the activity of key enzymes such as 1-aminocyclopropane-1-carboxylic acid (ACC) synthase and ACC oxidase, as has been observed under other metal stress conditions [51]. This suppression may impair stress mitigation, contributing to growth inhibition. Copper exposure likely induces oxidative stress in wheat seeds, contributing to cellular damage by generating ROS [52]. ROS can cause lipid peroxidation, protein denaturation, and DNA damage, which may disrupt normal cellular functions and lead to the growth retardation and developmental alterations observed [53]. Additionally, the inhibition of ethylene biosynthesis could amplify the stress response further, as ethylene is a critical hormone in mediating stress responses in plants. Ethylene overproduction has been linked to the induction of senescence, growth inhibition, and the activation of defense pathways under metal contamination [54,55,56].

A second major insight relates to the ammonia (NH_3_) accumulation, which increased significantly at 1 mM and 10 mM of CuSO_4_. This suggests the disruption of nitrogen metabolism [57], likely due to enhanced deamination of amino acids and the inhibition of glutamine synthetase, an enzyme responsible for ammonia detoxification [58,59]. Rising NH_3_ levels indicate a catabolic shift in metabolism, reflecting protein breakdown and impaired nitrogen assimilation under oxidative stress. The simultaneous suppression of ethylene and increase in ammonia emissions reflect a shift in the metabolic priorities under metal-induced stress and highlight the utility of CO_2_LPAS in capturing early-stage physiological perturbations in plants [60,61,62].

The statistical analysis using the one-way ANOVA and Tukey’s post hoc tests confirmed that both gas emissions were significantly altered by Cu exposure. Ethylene emissions were significantly reduced at all tested Cu concentrations (1 µM to 10 mM), while ammonia emissions increased significantly at concentrations of 100 µM and above. These findings point to robust, dose-dependent disruption of the primary metabolic pathways under Cu stress. The overall trends in ethylene and ammonia emissions observed across all CuSO_4_ treatments are further supported by the summary presented in Figure 10. The ethylene concentrations decreased consistently with increasing copper levels, reflecting dose-dependent inhibition of ethylene biosynthesis pathways. Conversely, ammonia emissions increased significantly only at concentrations of 100 µM and higher, indicating that low-level Cu exposure (1 µM) may not have yet disrupted nitrogen metabolism. The inclusion of the statistical annotations in Figure 10 offers a clear visual representation of these effects, which align closely with the numerical post hoc results originally detailed in Tables S1 and S2 (now moved to the Supplementary Materials for clarity). This dual trend—ethylene suppression and ammonia accumulation—provides robust evidence of distinct, concentration-dependent metabolic disruptions under copper stress during early seedling development. Our findings are relevant to plant physiology and significantly affect agricultural productivity and environmental health. Excess copper in soils due to using copper-based pesticides or industrial contamination poses a serious threat to crops, especially in regions with high environmental copper concentrations [63]. The effects of copper exposure observed in this study could lead to reduced wheat yields and altered grain quality, potentially impacting food security and economic stability in agricultural communities.

Moreover, while copper toxicity extends beyond wheat to other crops and plant species with similar sensitivities to heavy metal stress—with studies on cereals like maize and barley showing identical responses to copper exposure [64]—it is important to note that the direct findings of this study are specific to Triticum aestivum. Further research is necessary to confirm the precise mechanisms and dose-responses across a broader range of plant species and genotypes, as physiological responses to heavy metals can vary significantly.

Several studies have explored copper toxicity in a variety of plant species, revealing both shared and species-specific responses. In cucumber (*Cucumis sativus*), Alaoui-Sossé et al. [20] reported impaired carbohydrate metabolism and ion imbalances under excess copper, while in radish (*Raphanus sativus*), Lukatkin et al. [24] observed elevated oxidative stress markers without a clear correlation with ethylene signaling. In contrast, Filek et al. [36] demonstrated in wheat that radical pools were highly sensitive to oxidative agents such as selenium and zearalenone, supporting our EPR findings. Barley and maize, both cereal crops like wheat, show similar trends in photosynthetic inhibition under Cu stress [17,18], but relatively less is known about their real-time gas exchange responses.

What distinguishes the present study is its simultaneous monitoring of the ethylene and ammonia emissions over time combined with direct EPR evidence of radical suppression and copper complex formation in specific tissues.

EPR spectroscopy provided corroborating evidence of tissue-specific patterns of copper assimilation [65]. A consistent EPR signal (g = 2.0048) corresponding to semiquinone-type free radicals was detected in all samples, including untreated seeds. Upon exposure to Cu concentrations ≥ 1 mM, a secondary EPR signal attributed to Cu^2+^ complexes emerged.

In the root and agar samples, this Cu^2+^ signal was broad and lacked a resolved hyperfine structure, indicating cluster formation and strong exchange interactions. The suppression of the semiquinone signal at higher Cu levels suggests radical quenching or degradation by ROS. These EPR signatures are consistent with the findings of Filek et al. (2017) [36], who reported similar radical suppression and metal-induced signal broadening in wheat grains exposed to zearalenone and selenium. Our observations suggest that Cu^2+^, like other oxidative agents, can disrupt endogenous radical pools through ROS-mediated damage or direct metal–radical interactions.

In the grains, similar cluster formation was evident, and the Cu^2+^/radical intensity ratio increased nearly tenfold from 1 mM to 10 mM of CuSO_4_, indicating significant intracellular Cu uptake.

Copper exposure consistently induces oxidative stress by generating ROS and disturbing redox-active radical pools [66]; furthermore, tissue-specific EPR signatures indicate distinct detoxification or compartmentalization strategies in the roots, grains, and leaves, which have implications for plant resilience and copper tolerance [6,67]. Interestingly, the leaf tissues showed a distinct spectral profile. The Cu^2+^ signal exhibited well-resolved hyperfine splitting characteristic of isolated copper ions in a tetragonally distorted molecular environment. This suggests that unlike in the roots and grains, copper is more uniformly distributed in the leaves, likely due to differences in metal transport and compartmentalization mechanisms. The distinct EPR spectra in the leaf tissue echo the results from Labanowska et al. (2021), who observed differential responses in oat, barley, and wheat exposed to ozone [50]. Their data support the notion that leaves possess more robust detoxification and compartmentalization mechanisms compared to those in root systems. These findings support the use of tissue-specific strategies for copper detoxification and localization in plants.

These results have clear implications for both plant physiology and agricultural sustainability. Copper-based fungicides and industrial activities are known sources of soil contamination, and our data show that even micromolar levels of copper can disrupt hormonal and metabolic pathways in wheat seedlings. Ethylene suppression may impair growth and development, while ammonia accumulation signals metabolic stress and potential toxicity.

Furthermore, EPR spectroscopy revealed distinct tissue-specific patterns of copper uptake and radical suppression, advancing our understanding of Cu^2+^ detoxification strategies for different plant organs. In the roots and grains, EPR spectra showed broad, unresolved Cu^2+^ signals and a marked decrease in or the disappearance of semiquinone radicals at higher Cu concentrations, indicating Cu clustering and oxidative radical quenching. In contrast, the leaves exhibited well-resolved Cu^2+^ hyperfine structures, suggesting that copper remained in a more dispersed ionic state and was possibly compartmentalized better within the leaf tissues.

Together, these findings demonstrate that Cu^2+^ toxicity affects germinating wheat through a multifaceted mechanism involving (i) suppressed ethylene biosynthesis that hinders hormonal stress signaling; (ii) enhanced protein degradation and nitrogen dysregulation, and (iii) differential tissue responses to copper accumulation, indicating organ-specific detoxification and stress dynamics.

This mechanistic framework expands the current knowledge on metal-induced plant stress and shows that CO_2_LPAS and EPR can sensitively and dynamically capture these early stress signatures. These techniques not only provide non-destructive alternatives to the conventional methods but also enable real-time physiological monitoring with high specificity. A significant consideration is that this study was conducted under controlled indoor conditions. While these laboratory settings are crucial for isolating specific variables and ensuring reproducibility, they inherently do not fully replicate the complex environmental factors (e.g., varied soil composition, fluctuating temperatures, co-occurring stressors) encountered in natural agricultural fields. Therefore, validating these findings through future field-based studies is essential to understanding their implications under more realistic environmental scenarios.

The integrated application of CO_2_LPAS and EPR techniques successfully revealed how copper stress disrupts key physiological and biochemical processes in wheat seedlings. These findings not only expand our understanding of metal-induced stress mechanisms but also suggest practical diagnostic tools for early toxicity detection. However, a key limitation of this study is its focus on early-stage responses during germination. Future work should therefore investigate the long-term physiological impacts; examine the recovery mechanisms; and evaluate mitigation strategies, such as antioxidants and phytoremediation, for enhancing crop resilience to heavy metal stress. This approach could also be adapted to other stressors (e.g., cadmium, drought).

## 5. Conclusions

This study elucidates the early toxicological impact of copper exposure on germinating wheat seeds, integrating physiological and molecular markers to assess plant stress responses. Ethylene suppression and ammonia accumulation, detected via CO_2_ laser photoacoustic spectroscopy, reveal clear disruptions in hormonal signaling and nitrogen metabolism under Cu stress. Complementary EPR spectroscopy confirmed oxidative stress through the detection of semiquinone radicals and Cu^2+^ complexes, with tissue-specific patterns of copper accumulation and radical suppression.

The combined use of LPAS and EPR presents a powerful, non-invasive strategy for monitoring heavy metal toxicity in plants. These findings not only enhance our understanding of copper-induced stress mechanisms but also offer practical applications in environmental monitoring and sustainable agriculture. Future work should investigate the long-term physiological effects of metal stress and assess the efficacy of targeted mitigation strategies, such as antioxidants, genetic modification, and phytoremediation approaches, in enhancing plants’ resilience to copper and other heavy metals.

## Figures and Tables

**Table 1:** Selection of measurable factors for determining gases in common wheat seedlings.

Factor	Value/Description
Certified ethylene mixture for ethylene absorption coefficient measurement	0.96 ppm ethylene in pure nitrogen
Certified ammonia mixture for ammonia absorption coefficient measurement	10 ppm ammonia in pure nitrogen
Spectral range of the CO_2_ laser	9.2–10.8 µm
α (cm^−1^ atm^−1^)-gas absorption coefficient at a specific wavelength	V = *αCS*_M_*P*_L_*c*
Pressure in the OA cell	≅1030 mb
Gas absorption coefficient	α (C_2_H_4_) = 30.4 cm^−1^atm^−1^-10P(14) laser line/λ = 10.53 µm
	α (NH_3_) = 57.12 cm^−1^atm^−1^-9R(30) laser line/λ = 9.22 µm
Synthetic air composition	Linde gas: 20% oxygen, 80% nitrogen (impurities: hydrocarbons max. 0.1 ppm, nitrogen oxides max. 0.1 ppm)
Measurement temperature	23–25 °C
Responsivity of the FA cell	320 cmV/W
Volume of the FA cell	1000 cm^3^
Duration of one analysis	≅3600 s
Capacity of the KOH pellet enclosure	120 cm^3^

**Table 2:** Germination parameters.

Parameter	Value
CuSO_4_ solution concentrations (Sigma-Aldrich copper(II) sulfate pentahydrate (CuSO_4_·5H_2_O) with M = 249.99 g/mol)	1 µM, 100 µM, 1 mM, 10 mM
Dimensions of the Duran glass container used for germination	d = 48 mm, h = 40 mm
Washing and cleaning of the glass containers before germination	1% Alconox solution
Rinsing of the glass containers before germination	Distilled water
Number of common wheat seeds per container	40
Agar solution concentration	0.60%
Amount of agar solution per germination container	20 mL
Climate chamber temperature	22 °C
Climate chamber cycle for germination	Night
Amount of CuSO_4_ solution per container with common wheat seedlings	2.5 mL
Amount of distilled water per control sample container	2.5 mL
Number of samples per batch	3
Total number of samples with wheat seedlings	15
The period after which CuSO4 solutions were applied	72 h
Climate chamber cycle after CuSO4 solution application	Day/night
Respiration analysis	48 h after CuSO_4_ solutions were administered and 120 h after seeds were placed for germination
Duration of respiration measurements using LPAS	3 days

**Table 3:** The one-way ANOVA tests for ethylene (C_2_H_4_) and ammonia (NH_3_).

Gas	*p*-Value
Ethylene (C_2_H_4_)	*p* = 0.0054
Ammonia (NH_3_)	*p* = 0.0010

**Table 4:** Comparative tables and summaries for Days 1, 2, and 3.

CuSO_4_ Conc.	C_2_H_4_ (ppb)	% Change vs. DW	NH_3_ (ppb)	% Change vs. DW
		DAY 1		
DW	56.0	-	28.0	-
1 µM	28.5	−49.1%	26.0	−7.1%
100 µM	23.0	−58.9%	36.5	+30.4%
1 mM	17.5	−68.8%	41.0	+46.4%
10 mM	15.0	−73.2%	51.0	+82.1%
DW	56.0	-	28.0	-
		DAY 2		
DW	42.0	-	25.00	-
1 µM	19.0	−54.8%	38.0	+52.0%
100 µM	20.0	−52.4%	39.0	+56.0%
1 mM	15.7	−62.6%	46.0	+84.0%
10 mM	6.8	−83.8%	53.0	+112.0%
DW	42.0	-	25.00	-
		DAY 3		
DW	28.6	-	23	-
1 µM	14.0	−51.0%	40	+73.9%
100 µM	8.5	−70.3%	47	+104.3%
1 mM	14.0	−51.0%	55	+139.1%
10 mM	2.8	−90.2%	62	+169.6%

## Data Availability

The original contributions presented in this study are included in the article. Further inquiries can be directed to the corresponding authors.

## Supplementary Materials

The following supporting information can be downloaded at https://www.mdpi.com/article/10.3390/toxics13070604/s1. Table S1: Ethylene (C_2_H_4_)—Tukey HSD Summary; Table S2: Ammonia (NH_3_)—Tukey HSD Summary.
