# Supplementary material for: Evaluating Copper-Induced Oxidative Stress in Germinating Wheat Seeds Using Laser Photoacoustic Spectroscopy and EPR Techniques
Source: Toxics. 2025 Jul 18;13(7):604. doi: 10.3390/toxics13070604 (PMC12299222; doi:10.3390/toxics13070604)
Supplement: Supplementary file 1 [file toxics-13-00604-s001.zip › toxics-3739198-supplementary.pdf]

Tables S1 and S2 present the post-hoc Tukey HSD test results, showing which treatment groups differ significantly. All Cu treatments differ considerably from the control (DW) in ethylene emission. Ammonia levels vary significantly from the control, starting at 100 μM and above, but not at 1 μM.

Table S1. Ethylene (C_2H_4) – Tukey HSD Summary.

Group 1	Group 2	Mean Diff	p-adj	Lower	Upper	Reject?
DW	1 μM	24.80	0.008	5.35	44.25	Yes
DW	100 μM	29.97	0.003	10.52	49.42	Yes
DW	1 mM	33.80	0.001	14.35	53.25	Yes
DW	10 mM	43.13	0.000	23.68	62.58	Yes

Table S2. Ammonia (NH_3) – Tukey HSD Summary

Group 1	Group 2	Mean Diff	p-adj	Lower	Upper	Reject?
DW	1 μM	9.33	0.058	-0.11	18.78	No
DW	100 μM	15.83	0.001	6.38	25.28	Yes
DW	1 mM	20.00	0.000	10.55	29.45	Yes
DW	10 mM	26.67	0.000	17.22	36.12	Yes
